# Dietary Milk or Isolated Legume Proteins Modulate Intestinal Microbiota Composition in Rats

**DOI:** 10.3390/nu16010149

**Published:** 2024-01-02

**Authors:** Luis A. Rubio

**Affiliations:** Department of Animal Nutrition and Sustainable Production, Estación Experimental del Zaidin (CSIC), Profesor Abareda 1, 18008 Granada, Spain; lrubio@eez.csic.es; Tel.: +34-958-572757; Fax: +34-958-572753

**Keywords:** intestinal microbiota composition, lactalbumin, casein, *Cicer arietinum*, *Lupinus angustifiolius*, protein isolates, rat

## Abstract

Shifts toward increased proteolytic fermentation, such as, for example, in athlete and high-protein weight loss diets, may alter the relative abundance of microbial species in the gut and generate bioactive, potentially deleterious metabolic products. In the current investigation, intestinal (caecal) microbiota composition was studied in rats fed diets differing only in their constituent proteins: milk (casein (CAS), lactalbumin (LA)) or legume (*Cicer arietinum, Lupinus angustifolius*) protein isolates (chickpea protein isolate (CPI), lupin protein isolate (LI)). ANOSIM and Discriminant Analysis showed significant (*p* < 0.05) differences at both family and genus levels in both microbiota composition and functionality as a consequence of feeding the different proteins. Differences were also significant (*p* < 0.05) for predicted functionality parameters as determined by PICRUSt analysis. LA induced a generally healthier microbiota composition than CAS, and higher amounts of *Methanobrevibacter* spp. and Methanogenic_PWY were found in the LI group. LEfSe analysis of bacterial composition and functional activities revealed a number of groups/functions able to explain the different effects found with milk and legume protein isolates. In conclusion, the mostly beneficial modulation of intestinal microbiota generally found with legume-based diets is likely to be due, at least in part, to their constituent proteins.

## 1. Introduction

Dietary protein accounts for up to 30% (70–100 g of protein/day) of the human diet and has a wide range of nutritional and biological functions. However, excess protein, together with peptides and free amino acids (AAs) that escape absorption of the small intestine, enter the large intestine and is fermented by the resident microbiota. Protein has received increasing attention among the various food nutrients also because the distal colon is the primary site of protein fermentation where many toxic substances, such as hydrogen sulfide, ammonia, and a series of phenolic and indolic compounds detrimental to health and implicated in colon cancer and bowel disease, are produced [1].

Shifts toward increased proteolytic fermentation (such as, for example, in athlete and high-protein weight loss diets, where protein intake may be two to five times greater than the daily dietary recommendations) may alter the relative abundance of microbial species in the gut and generate bioactive, potentially deleterious metabolic products. These metabolic products have been shown to increase inflammatory response, tissue permeability, and colitis severity in the gut. They are also implicated in the development of metabolic diseases, including colorectal cancer, obesity, diabetes, and nonalcoholic fatty liver disease [2]. To offset the negative net muscle protein balance in athletes under weight loss diets, strategies, such as increasing protein intake, are recommended. There is some evidence to support the role of whey, as a high-quality protein, in the promotion of high-quality weight loss during caloric restriction, and alpha-lactalbumin is one of the main components of whey protein [3]. However, the information on the effects that modifications of the dietary protein quality and/or quantity may have on the digestive physiology, and in particular on the intestinal microbiota composition/functionality, is still very limited. On the other hand, isolated legume storage proteins have been previously reported to be readily digested within the small intestine and individual AAs digestibility of these proteins was high. However, previous work showed that under normal feeding conditions AAs from CPI and LI are absorbed at slower rates than those from animal proteins, which might explain the lower nutritional utilization of legume storage proteins as compared with LA or CAS [4]. Lower rates of AAs portal absorption and arterial plasma in rats fed legume proteins may also contribute to a lower risk of processes such as, for example, heart failure, which has been linked to high plasma BCAAs (branch chain amino acids: valine, isoleucine, leucine) levels [5].

Future research challenges in this area of research are to identify bacteria affected by diet, food components, foods or dietary patterns that have an impact on the microbiota, and microbes that are key players in mediating the health effects of different dietary components. That information can be used to design successful dietary strategies to benefit health. In this context, many reasons advise a more detailed study of the effects of the type or amount of dietary protein on intestinal microbiota composition or metabolism. Thus, being proteins one of the major components of any daily healthy diet, and apart from nutritional considerations (what AAs and at what rate they are absorbed, etc.), as already mentioned, many of the compounds released after bacterial protein degradation are known to be toxic and detrimental to gut health [6]. In addition, metabolic cross-feeding is a central feature in anaerobic microbial communities [7], so the products of the fermentation from a given group may influence the growth/metabolism of other groups. A review focusing on current insights into changes associated with dietary protein-induced gut microbial populations and their potential roles in the metabolism, health, and disease of animals has been recently published [8].

It is currently well established that the composition of the microbial communities inhabiting the human intestine has important consequences for health, and their composition and activities are known to be strongly influenced by the diet, which has long been considered the major external modulator of the adult human intestinal microbiota in parallel with medication. In particular, microbial composition and functional activities are known to be strongly influenced by the carbohydrate content of the diet, mainly because the human digestive system cannot digest most of the plant-derived complex carbohydrates present in a normal diet, while our intestinal bacteria encode an arsenal of catabolic enzymes to degrade and ferment a wide range of polysaccharides and glycans of dietary or host origin that enter the colon. However, the colonic fermentation of protein has received much less attention than that of carbohydrates [9].

Accordingly, energy- and protein-equalized semisynthetic diets, which differed only in the composition of their constituent proteins (LA, CAS, CPI, LI), were produced to study the effects on caecal microbiota composition in rats. Diets were not supplemented with lacking essential AAs so that only the effect of the proteins themselves was measured.

## 2. Materials and Methods

### 2.1. Protein Purification

Chickpea (*Cicer arietinum*, kabuli var.) and lupin (*Lupinus angustifolius*, sweet var.) seeds were purchased locally. Protein isolates were obtained from defatted chickpea and lupin seed meals by acid precipitation at the isoelectric point, as previously described [10].

### 2.2. Animals and Diets

LA, casein and chemical reagents used were from Sigma Chemical Co. (Alcobendas, Madrid, Spain). The diets used were based on animal proteins (LA, CAS) or legume protein isolates (CPI and LI) and contained the same amounts of digestible energy (15.5 kJ/g) and protein (150 g/Kg). Proteins were added at the expense of maize starch so that all of them had the same composition, except for differences due to amounts of AAs in each particular protein. The diets contained (g/Kg) protein (150), maize starch (450), potato starch (150), sunflower oil (50), glucose (150), and a vitamins and minerals mix (100) to meet requirements [10]. AAs composition of the proteins used is shown in Table 1. Male weaned Wistar rats (*n* = 6 per treatment), matched by weight (130 ± 1.5 g), were housed individually in metabolism cages under controlled conditions of temperature (25 °C), moisture (50%) and lighting (12 h cycles). Animals were fed a control AINS93 diet and then their respective experimental diets for 5 d before the day of sampling. Water was freely available at all times. Rats were killed under pentobarbital sodium (40 mg·Kg^−1^ BW) anesthesia exactly 3 h after ingesting 4 g of feed, the abdomen opened, and the caecal contents were extracted and stored for less than 7 d at −20 °C until freeze-dried (Genesys SQ25EL lyophiliser, VirTisCo, New York, NY, USA). Lyophilization has been shown to improve both DNA yield and the quality of the information arising from the PCR–RFLP method of analysis [11].

The experimental protocol was reviewed and approved by the Institutional Animal Care and Use Committee of the Spanish Council for Scientific Research (CSIC, Spain), and the animals cared for in accordance with the Spanish Ministry of Agriculture guidelines (RD 53/2013).

### 2.3. RT-qPCR Microbiota Composition Analysis

Total DNA was isolated from freeze-dried caecal samples (40 mg) using the FavorPrep Stool DNA Isolation Mini Kit (Favorgen-Europe, Vienna, Austria) and following the manufacturer’s instructions. Eluted DNA was treated with Rnase and the DNA concentration was assessed spectrophotometrically by using a NanoDrop ND-100 Spectrophotometer (NanoDrop Technologies, Wilmington, DE, USA). Purified DNA samples were stored at −20 °C until use. Eluted DNA was treated with Rnase and the DNA concentration was assessed by using a NanoDrop ND-100 spectrophotometer (NanoDrop Technologies, Wilmington, DE, USA). Bacterial log_10_ number of copies was determined by using quantitative polymerase chain reaction (q-PCR) (iQ5 Cycler, Bio-Rad Laboratories, Alcobendas, Spain). The 16S rRNA gene-targeted primers and polymerase chain reaction (PCR) conditions used in this study were as described previously [14]. The different microbial groups quantified included *Lactobacillus* spp., *Bifidobacterium* spp., *Blautia coccoides/Eubacterium rectale* group, *Clostridium leptum/Ruminococcus* spp., *Enterobacteriaceae*, *Escherichia/Shigella*, *Bacteroides/Prevotella* spp., and total bacteria. Samples for q-PCR analysis were run in duplicate.

### 2.4. High-Throughput Analysis of Microbial Community

The bacterial diversity of the samples was determined using Illumina technology (MiSeq, Illumina Centre, Cambridgeshire, UK). Total DNA was isolated from freeze-dried caecal samples (40 mg) from five rats per group (*n* = 20) as described above. Libraries preparation was performed by amplification of the V4–V5 region of the 16S rRNA gene by using a Px2 Thermal Cycler (Thermo Electron Corporation, Waltham, MA, USA). The first amplification was performed by using primers Mi_U515 (50-GTGCCAGCMGCCGCGGTAA-30) and Mi_E786 (50–GGACTACHVGGGTWTCTAAT-30) including partially Illumina adapters Mi_E786 (50–GGACTACHVGGGTWTCTAAT-30), and PCR conditions were initial denaturalization at 98 °C 30 s, 25 cycles with denaturalization at 98 °C 10 s, annealing at 52 °C 20 s and extension at 72 °C 10 s, and a final extension at 72 °C 5 min. The second amplification included barcodes and the rest of the Illumina adapters, and PCR conditions were initial denaturalization at 98 °C 30 s, 25 cycles with denaturalization at 98 °C 10 s, annealing at 52 °C 20 s and extension at 72 °C 10 s, and a final extension at 72 °C 5 min. All amplifications were performed in duplicate. Total DNA was isolated from freeze-dried feces (20 mg) as described above. Aliquots of 10 μL of each DNA were sent to the IPBLN (CSIC, Granada, Spain) for sequencing.

### 2.5. Analyses of Predicted Microbial Functions

Functional gene compositions of bacterial communities were predicted using the PICRUSt (phylogenetic investigation of communities by reconstruction of unobserved states) method [15]. To generate BIOM-formatted files for PICRUSt input data, taxonomic classification was re-processed in QIIME2 version 2021.11 with the GreenGenes V13.8 database [16]. PICRUSt functionality was run using the QIIME2-produced biom files via the QIIME2. Functional prediction was made using the KEGG orthologs database [17] and summarized at the pathway hierarchy level 3.

### 2.6. Statistical Analysis

Results other than sequencing analysis were subjected to one-way ANOVA and Tukey’s multiple comparison test for differences between means [18]. Significance was established at *p* < 0.05. Results from high throughput sequencing analyses were obtained by using Quantitative Insights in Microbial Ecology (QIIME2 2021.11). Quality filtering was performed by using default parameters in QIIME2. A sub-OTU (suboperational taxonomic units) table in biom format was created using Deblur, and alignment and taxonomic assignation was performed by fragment insertion script and against the Greengenes database. Sub-OTUs obtained by Illumina analysis of caecal samples from 24 rats (six per treatment) were grouped by bacterial species (obtained from the bar plots produced by QIIME2). Multivariate statistical techniques explored the similarities in rat caecal microbiota and identified species accounting for differences observed in these bacterial communities. Bray–Curtis measures of similarity were calculated to examine similarities between gut microbial communities of rats from the high throughput and qPCR data matrices, following standardization, and square-root transformation. The Bray–Curtis similarity coefficient is a reliable measure for biological data on community structure and is not affected by joint absences, which are commonly found in microbial data. Analysis of similarity (ANOSIM) was performed to test whether gut microbial communities were significantly different between treatments. Analysis of similarity percentages (SIMPER) was done to determine the overall average similarity in caecal microbial community compositions. Discriminant analysis (DA) was used to check if the groups to which observations belong were distinct. Statistical tests for differentially abundant families, genera, and functional categories were performed using the linear discriminant analysis effect size (LEfSe) method [19] with an alpha value of 0.05 for the Kruskal–Wallis test among classes, and the threshold for the log_10_LDA score was set at 2.0. Microbial functions were predicted using Phylogenetic Investigation of Communities by Reconstruction of Unobserved States (PICRUSt), based on high-quality sequences [15]. OTUs were normalized by copy number, and metagenomic prediction was performed based on Kyoto Encyclopedia of Genes and Genomes (KEGG) [17] by using the QIIME2 version 2021.11 package. The Simpson (D), Shannon index (H), Evenness (E) and Chao1 (Chao1) indexes of the bacterial community were respectively calculated as:

D = 1/(Σ s i = 1pi2)

where s is the total number of species in the community and pi is the proportion of community represented by OTU i

H = −Σ (pi ln pi)

where pi is the abundance of each species.

E = H/lnS

where S is the total number of species.

Chao1 = Sobs + [F1(F1 − 1)/2(F2 + 1)]

where F1 and F2 are the count of singletons and doubletons, respectively, and Sobs is the number of observed species.

## 3. Results

### 3.1. RT-qPCR Microbiota Composition Analysis

**Figure 1:**
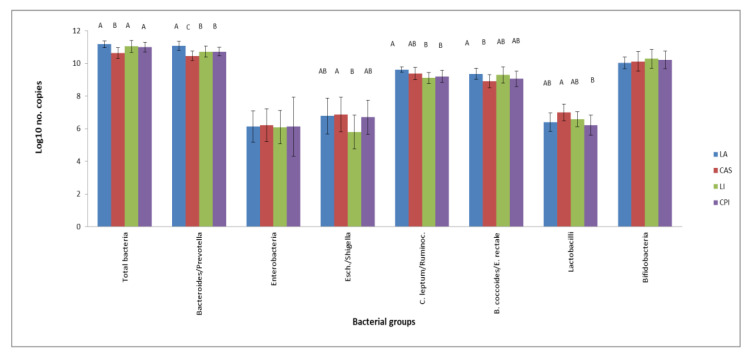
RT-qPCR bacterial counts (log_10_ copies of the 16 S-rRNA gene mg-1 dry content) in the caecal content of rats fed based on milk (LA, CAS) or legume protein isolates (CPI, LI) as the only protein source. Values are means (*n* = 6) with SD in bars. Different letters indicate significant (*p* < 0.05) differences.

Results on caecal bacteria numbers after consumption of the experimental diets were collected in Figure 1. The CAS diet induced lower total bacteria and *Bacteroides/Prevotella* compared to the other groups and lower *B. coccoides/E. rectale* numbers with respect to the LA group. CPI and LI diets gave place to lower *Bacteroides/Prevotella* and *C. leptum/Ruminococcus* with respect to LA, but values for *Bacteroides/Prevotella* were higher than CAS. Rats fed the LI diet had lower *Escherichia/Shigella* numbers compared to the CAS diet.

### 3.2. High-Throughput Analysis of Microbial Community

A total of 3,149,558 reads were obtained from the 24 caecal samples processed through Illumina MiSeq technology. After Deblur, 2,548,825 good-quality sequences belonging to 1482 OTUs and 108 bacterial species were retained for subsequent analyses. A similarity percentages breakdown (SIMPER analysis) (Table S1) was used to select those bacterial groups with higher contribution to dissimilarity. Thus, 22 families (*Bacteroidaceae, Spirochaetaceae, Lachnospiraceae, Bacteroidales* families, *Ruminococcaceae, [Paraprevotellaceae], Bifidobacteriaceae, Prevotellaceae, Clostridiales* families, *Helicobacteraceae, Bacteroidales* families, *Erysipelotrichaceae, Clostridiaceae, Methanobacteriaceae, Lactobacillaceae, Veillonellaceae, Alphaproteobacteria families, Chloroflexi* families, *Alcaligenaceae, Cyanobacteria* families, *Porphyromonadaceae* and *Enterobacteriaceae*), and 27 genera (*Treponema, Bacteroidales* genera, *Ruminococcus, Lachnospiraceae* genera, *Bacteroides, [Prevotella], Bifidobacterium*, *Ruminococcaceae* genera, *Roseburia, Helicobacter, Allobaculum, Clostridiales* genera, *Bacteroidales* genera, *Phascolarctobacterium, Lactobacillus, Prevotella, Chloroflexi* genera, *Alphaproteobacteria* genera, *Sutterella, Helicobacteraceae* genera, *Blautia, Coprococcus, Oscillospira, Methanobrevibacter, Parabacteroides* and *Cyanobacteria* genera) were responsible for >95% of the dissimilarity.

**Figure 2:**
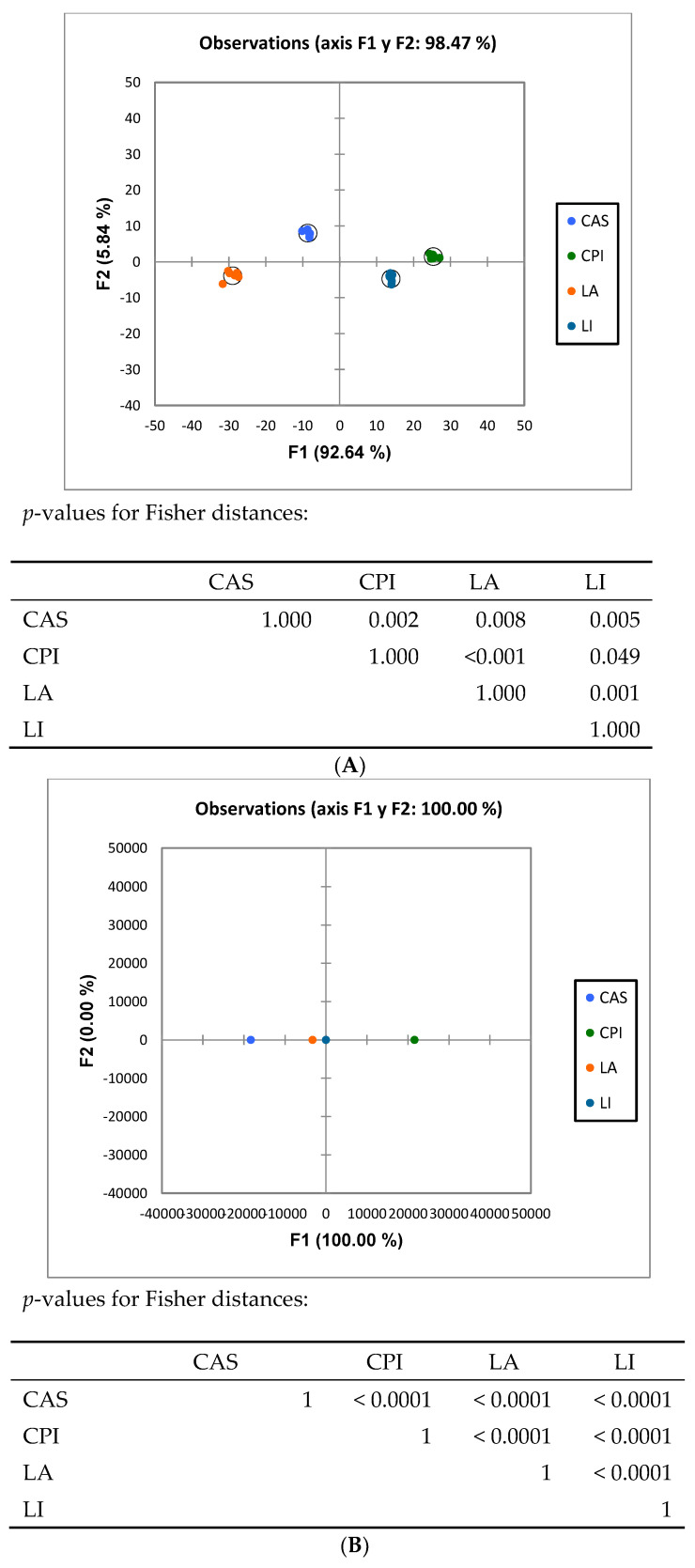
Discriminant Analysis of bacterial groups analyzed by Illumina sequencing. LA, la ctalbumin; CA S, casein; CPI, chickpea protein isolate; LI, lupin protein isolate. (**A**) Family level, (**B**) genera level.

ANOSIM analysis (Table 2) analysis of the high throughput results showed that the caecal microbiota composition in rats fed the different diets was different (*p* < 0.01) in all cases. Discriminant analysis (Figure 2) of the pyrosequencing results showed that the rats fed the diets differing in the type of protein were grouped differently at both the family and genus levels.

*Bacteroidetes, Firmicutes, Proteobacteria, Spirochaetes* and *Actinobacteria* were the most abundant phyla in all treatments (Table 3), although *Chloroflexi, Cyanobacteria* and *Tenericutes* were also found at the different taxonomic levels. Interestingly, *Euryarchaeota* belonging to the *Archaea* domain was particularly abundant in the LI group. Significant differences (*p* < 0.05) were found for all phyla except for *Actinobacteria*. At the family level (Table 3), *Ruminococcaceae* (20.50%), families from *Bacteroidales* (19.24%), *Spirochaetaceae* (11.33%), *Bacteroidaceae* (9.94%) and *Lachnospiraceae* (9.65%) were generally the most abundant. In particular, *Bacteroidaceae* and *Clostridiaceae* were the most abundant (*p* < 0.05) in rats fed the LA diet. Rats fed the CAS diet had higher (*p* < 0.05) *Ruminococcaceae*, *Veillonellaceae* and *Helicobacteraceae* reads, and *Bifidobacteriaceae* tended to be higher. Feeding the CPI diet induced higher *Prevotellaceae* than the other groups, while families from *Clostridiales* and *Spirochaetaceae* were higher in CPI and LI diets with respect to LA and CAS diets. LI diet gave place to higher (*p* < 0.05) *Methanobacteriaceae* and *Lachnospiraceae* compared to the other groups.

At the genera level (Table 3), *Bacteroides, Blautia* (except for CPI) and *Roseburia* were higher (*p* < 0.05) in LA than in the other groups, while *Treponema* was lower (*p* < 0.05). Rats fed the CAS diet had higher (*p* < 0.05) *Ruminococcus, Phascolarctobacterium* and *Helicobacter* reads, and *Bifidobacterium* tended to be higher. Feeding the CPI and LI diets induced higher (*p* < 0.05) *Prevotella* and genera from the *Clostridiales* than the LA and CAS groups. The LI diet gave place to higher (*p* < 0.05) *Methanobrevibacter* values compared to the other groups. The species identified within these genera were *Bacteroides uniformis, Balutia producta, Roseburia faecis*, *Ruminococcus gnavus, R. bromii* and *R. flavefaciens, Prevotella copri, Bifidobacterium animalis* and *B. pseudolongum*.

**Figure 3:**
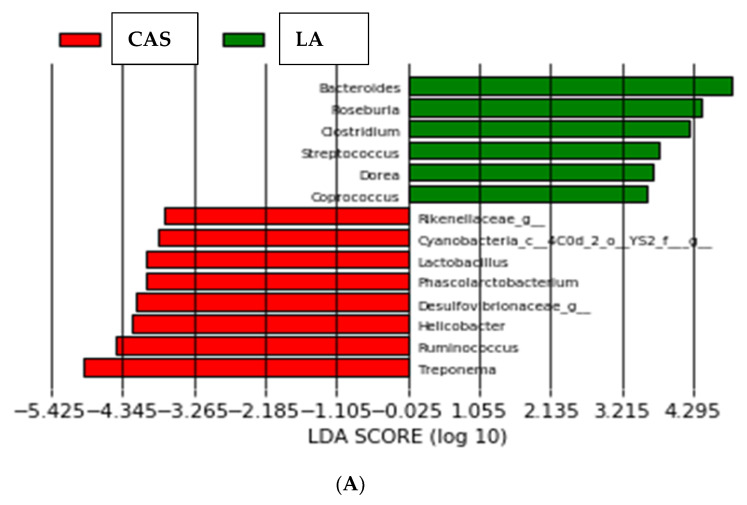

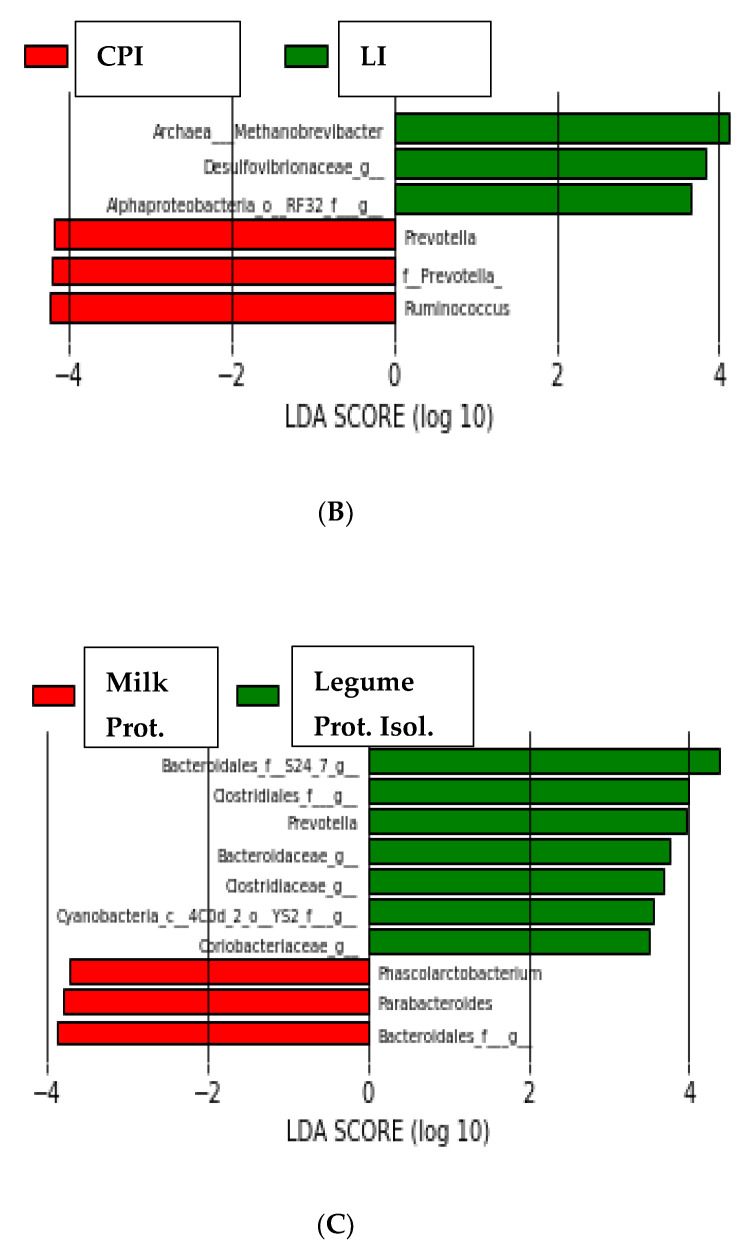
Linear discriminant analysis coupled with effect size (LEfSe) of bacterial groups after SEMPER analysis, using the default parameters (LDA score = 2). (**A**) Different taxa between CAS and LA. (**B**) Different taxa between CPI and LI. (**C**) Different taxa between animal (milk) and vegetable (legume) proteins.

LEfSe determines the features (organisms, clades, operational taxonomic units, genes, or functions) most likely to explain differences between classes by coupling standard tests for statistical significance with additional tests encoding biological consistency and effect relevance [20]. As shown in Figure 3A, the organisms most likely to explain differences between LA and CAS were *Bacteroides* spp., *Roseburia* spp., *Clostridium* spp., *Streptococcus* spp., *Dorea* spp. and *Coprococccus* spp. for the LA group; *Treponema* spp., *Ruminococcus* spp., *Helicobacter* spp., genera from *Desulfovibrionaceae*, *Phascolarctobacterium, Lactobacillus* spp., genera from *Cyanobacteria* and genera from *Rikenellaceae* for the CAS group. The organisms most likely to explain differences between CPI and LI (Figure 3B) were *Ruminococcus* spp., genera from *Prevotella* and *Prevotella* spp. for CPI; *Methanobrevibacter* spp. (*Archaea*), genera from *Desufovibrionaceae* and genera from *Alphaproteobacteria* for LI. Finally, the organisms most likely to explain differences between animal (milk) and vegetable (legume) proteins (Figure 3C) were genera from the following order:

*Bacteroidales*, *Parabacteroides* spp. and *Phascolarctobacterium* spp. for the animal proteins; genera from the family S24_7, genera from the order *Clostridiales*, *Prevotella* spp., genera from *Bacteroidaceae*, *Clostridiaceae*, *Cyanobacteria* and *Coriobacteriaceae* for the vegetable proteins.

**Figure 4:**
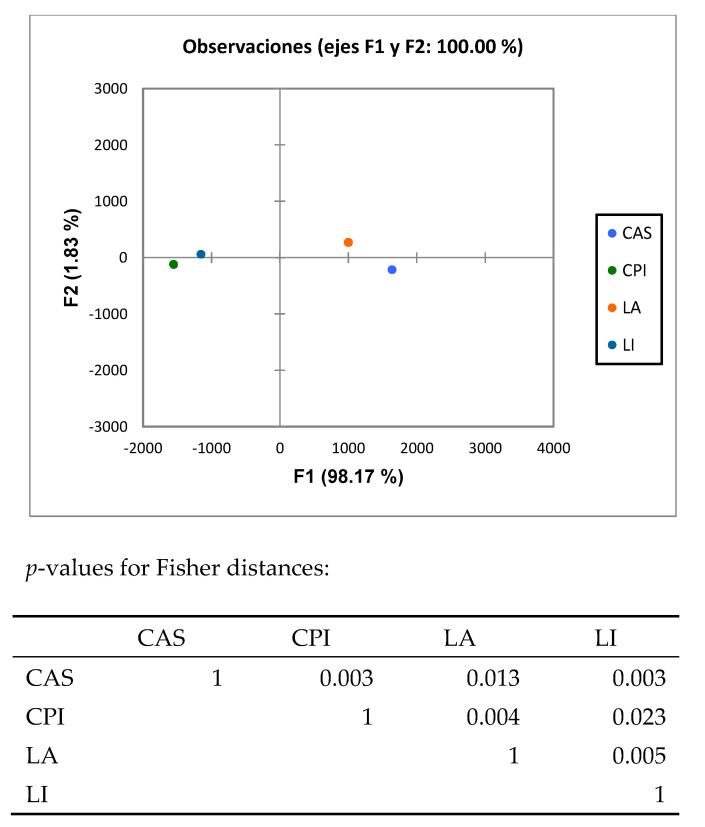
Discriminant analysis of predicted functions (PICRUSt analysis) of the intestinal microbiota bacterial groups analyzed by Illumina sequencing; LA, lactalbumin; CAS, casein; CPI, chickpea protein isolate; LI, lupin protein isolate.

**Figure 5:**
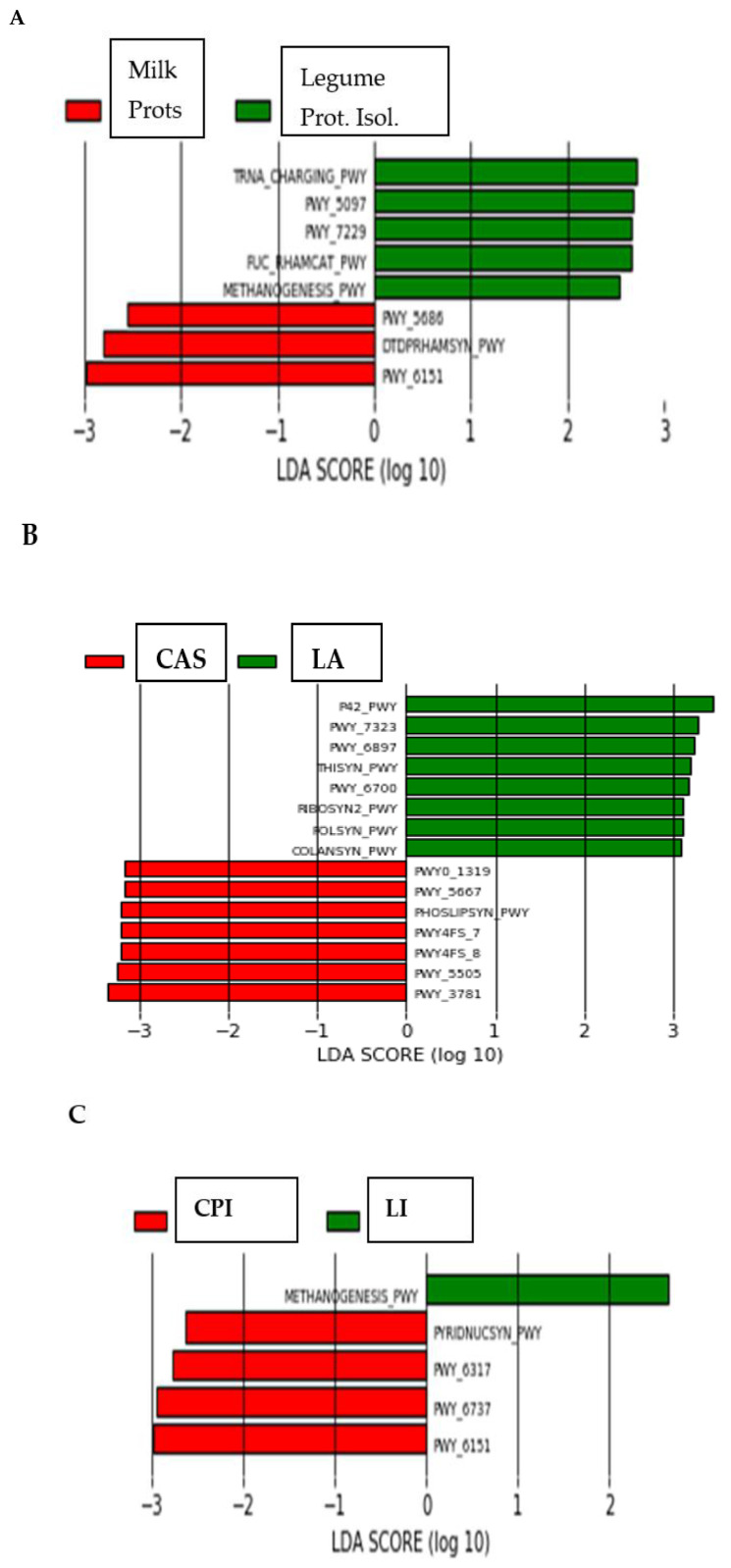
Linear discriminant analysis coupled with effect size (LEfSe) of predicted functions (PICRUSt analysis) after SIMPER analysis, using the default parameters (LDA score = 2). (**A**) Different taxa between animal (milk) and vegetable (legume) proteins. (**B**) Different taxa between CAS and LA. (**C**) Different taxa between CPI and LI.

The predicted functions of the intestinal microbiota were identified by using PICRUSt. Discriminant analysis (Figure 4) of the PICRUSt analysis showed that the functionality of the intestinal microbiome in rats fed the diets differing in the type of protein differed significantly (*p* < 0.05). Also, most functions of the caecal microbiota were differentially enriched in the LA group (Figure S1). The exceptions were Methanogenesis_PWY, which was enriched in the LI group, and PWY_3781, which was enriched in the CAS group. The LEfSe analysis of functions (Figure 5) showed that out of the 388 identified functions, 5 functions (TRNA_charging_PWY, PWY_5097, PWY_7229, FUC_rhamcat_PWY and Methanogenesis_PWY) discriminated the vegetable (CPI and LI isolates) and 3 functions (PWY_6151, Dtdprhamsyn_PWY and PWY_5686) discriminated the animal (milk) proteins. Seven (7) and 8 functions explained differences between the CAS (PWY_3781, PWY_5505, PWY4FS_7, Phoslipsin_PWY, PWY_5667 and PWY0_1319), and LA (P42_PWY, PWY_7323, PWY_6897, Thisyn_PWY, PWY_6700, Ribosyn2_PWY, Folsyn_PWY and Colansyn_PWY) diets, respectively. Methanogenesis_PWY specifically differentiated the LI diet, and PWY_6151, PWY_6737, PWY_6317 and Piridnucsyn_PWY differentiated the CPI diet.

Regarding the diversity indexes (Table 4), there were no differences for Evenness at the family level and Simpson and Evenness at the genus level. The Simpson, Shannon and Chao1 indexes at the family level and Shannon and Chao1 at the genus level were lower (*p* < 0.01) for the LA diet. At the family level, Simpson, Shannon and Chao1 indexes for the LI diet were higher (*p* < 0.01) than LA and CAS, but not different from CPI. Shannon and Chao1 at the genus level were higher (*p* < 0.01) for LI compared to the LA diet.

## 4. Discussion

Increased proteolytic fermentation in the gut may alter the relative abundance of microbial species in the gut and generate bioactive, potentially deleterious metabolic products. However, published work specifically aimed to compare in a systematic way the effects induced in vivo by chemically defined proteins on the intestinal microbiota composition and function, without the interference of other dietary components, is as yet quite scarce. To that end, semisynthetic diets for rats differing only in their constituent proteins were formulated. The proteins utilized here were two of animal (milk) origin (LA, CAS) and two of vegetable (legume) origin (CPI, LI). Both qPCR and Illumina sequencing analysis revealed significant differences in composition and functionality as a consequence of feeding different dietary proteins. Thus, both ANOSIM and Discriminant Analysis (Table 2 and Figure 2) elicited very significant differences among the different dietary groups at both family and genus levels. Results from qPCR on microbiota composition were in line with those from sequencing. See, for example, the higher *Bacteroides* spp. and *Blautia* spp. values found in LA as compared to the CAS diet (Figure 1 and Table 3). As for the variability indexes, at the family and genus levels, the results pointed to differences mainly in the richness indices (Shannon, Chao1) more than in evenness indices (Evenness, Simpson) (Table 4). The main effect observed was lower (*p* < 0.05) richness values for the LA diet as compared to the other groups including CAS. So, the number of different species inhabiting the LA caecal contents was lower, while the uniformity of the microbial populations was similar between treatments. This is interesting in connection with the results obtained on functional analysis (PICRUSt) (see below).

At the family level, *Bacteroidaceae* and *Clostridiaceae* were the most abundant (*p* < 0.05) in rats fed the LA diet (Table 2). The main genera represented were *Bacteroides* (mainly *Bacteroides uniformis*), *Blautia* (mainly *Blautia producta*) and *Roseburia* (mainly *Roseburia faecis*). Interestingly, *Treponema* spp. were much lower (*p* < 0.0001) in the LA diet than in the other diets including CAS. Rats fed the CAS diet had higher (*p* < 0.05) *Ruminococcaceae*, *Veillonellaceae* and *Helicobacteraceae* reads, while *Bifidobacteriaceae* tended to be higher. The main genera represented were *Ruminococcus* (mainly *R. gnavus, R. bromii* and *R. flavefaciens*), *Phascolarctobacterium* and *Helicobacter*. *Bifidobacterium* (mainly *Bifidobacterium animalis* and *B. pseudolongum*) also tended to be higher. Feeding the CPI diet induced higher *Prevotellaceae* than the other groups and higher *Methanobacteriaceae* in the LI group, while families from *Clostridiales* and *Spirochaetaceae* were higher in CPI and LI diets with respect to LA and CAS diets. The main genera represented were *Methanobrevibacter, Prevotella* (mainly *P. copri*) and genera from the *Clostridiales* order. *Parabacteroides* spp. was higher than LI in LA and CAS diets but not different from CAS.

Growing evidence has indicated that AAs, the main products of dietary protein digestion, can affect the structure, composition, and functionality of gut microbiota. It is well known that a number of species are implicated in proteolytic fermentation in vitro, and include bacteria from the genera *Clostridium, Fusobacterium, Bacteroides, Actinomyces, Propionibacterium* and *Peptostreptococci* [20]. However, the species with the greatest capacity for proteolytic fermentation in vivo cannot be identified just in a noncompetitive in vitro environment but would require a model closer to the highly competitive intestinal environment. In addition, due to the differences in substrate abundance, community membership, and species richness in different locations of the gut, it is important not only to establish which species are participating but also to examine how processes may differ in the small and large intestines [2]. It has been reported that several microbial groups, such as *Bacteroidetes, Actinobacteria, Firmicutes, Proteobacteria* and *Verrucomicrobia*, and genera, such as *Roseburia* and *Lactobacillus*, are sensitive to peptides that result in changes in the composition and diversity of gut microbiota [21,22]. Except for *Verrucomicrobia*, the results found here (Table 3) are in line with these previous reports, as these mentioned microbial groups are among those significantly affected by the different diets.

LEfSe analysis (Figure 3) revealed, on the other hand, that the organisms most likely to explain differences between LA and CAS were *Bacteroides* spp., *Roseburia* spp., *Clostridium* spp., *Streptococcus* spp., *Dorea* spp. and *Coprococcus* spp. for the LA group; *Treponema* spp., *Ruminococcus* spp., *Helicobacter* spp., genera from *Desulfovibrionaceae*, *Phascolarctobacterium, Lactobacillus*, and genera from *Cyanobacteria* and *Rikenellaceae* for the CAS group. Most of the organisms pointed out by LEfSe for the LA group (namely *Bacteroides* spp., *Roseburia* spp., *Clostridium* spp., and *Coprococcus* spp.) have been identified as butyrate producers [23,24]. The three major SCFAs (acetate, propionate and butyrate) are the main end products from carbohydrate fermentation in the proximal colon but are also produced from AAs in the distal colon. Among the SCFAs, butyrate is the main energy source for epithelial cells, as 70–90% is metabolized in the colonocytes. Branched-chain fatty acids (BCFAs, i.e., isobutyrate, 2-methybutyrate and isovalerate), which also belong to SCFAs and represent between 5 and 10% of total SCFAs are originated by the microbiota exclusively from BCAAs. SCFAs supply intestinal epithelial cells with energy, exert anti-inflammatory effects and regulate metabolism through binding to G-protein coupled receptors. Production of SCFAs lowers the luminal pH of the colon, which markedly affects the composition of the colonic microbiota by preventing overgrowth of pH-sensitive pathogenic bacteria such as Escherichia and some *Clostridia* [1]. On the contrary, a number of species from some of the groups pointed out for CAS (*Treponema* spp., *Helicobacter* spp., and genera from *Rikenellaceae*) have been reported as pathogenic. Thus, for example, some *Treponema* spp. as *Treponema pallidum* (Phylum *Spirochaetota*) is known to be responsible for diseases such as syphilis [24]; *Helicobacter pylori* is the main agent in peptic ulcer disease [25]; and members of the *Rikenellaceae* family have been implicated in both beneficial [26] and pathological processes (colorectal cancer) [27]. Even more, *Treponema* spp. were much lower in the LA diet than in the other diets including CAS. Thus, according to the current research, and in the absence of other dietary components, LA appears to induce a generally healthier microbiota composition than CAS and would then be preferable in high-protein diets.

There are three situations where high protein intake is most commonly utilized: in muscle hypertrophy, particularly among bodybuilders, powerlifters and other strength athletes; during energy-restricted weight loss diets; and in recovery from intense exercise. Apart from other metabolic risks advocated for high protein diets (renal dysfunction, loss of bone mass, atherogenesis), the main potential problem with this type of diet is the substitution of proteins for other macronutrients, particularly carbohydrates [28]. This is relevant in the present context because the proportion of protein to carbohydrate contents in the diet is usually regarded as the main driver of intestinal microbiota composition [9] and, as already mentioned, SCFAs (the main energy source for epithelial cells) are the main end products from carbohydrates fermentation in the proximal colon [29]. Therefore, high-protein diets are likely to have direct implications due to the composition of the proteins themselves and also indirect consequences as a result of changes in the proportions of other dietary components, particularly carbohydrates.

Piglets with highly digestible casein-based diets have been shown to present a higher count of *Enterobacteriaceae* than piglets fed on less digestible soybean meal-based diets [30]. This is in agreement with previous work by our group [31], where the number of copies in animals fed a casein-based diet was lower than soybean for Lactobacilli and *Bacteroides*, but was higher than soybean for *Bifidobacteria, Enterobacteria* and the *Escherichia/Shigella* group. However, since pure proteins were not used in diet formulation in these and most other reports, the main difficulty in ascribing these effects to the protein component of the diets mainly lies in the fact that they also differed in the carbohydrate fraction. The dietary proportion of protein to carbohydrate contents is usually regarded as the main driver of intestinal microbiota composition [9]. Thus, for example, the proportion of the families *Lachnospiraceae* and *Ruminococcaceae* were decreased, while the proportions of the genus *Bacteroides* and *Parabacteroides* were increased in mice fed with a high-protein and low-carbohydrate diet, which may result in a deleterious gut environment [32]. However, as other components, such as polyphenols, are known to have a substantial effect [9], it is necessary to establish what effects are mainly or only related to the protein fraction of the diet.

As for the legume PIs, the LI diet resulted in lower qPCR *Escherichia/Shigella* values than LA and CAS (Figure 1). This is in agreement with previous work by our group, where lower *Enterobacteria* and *Escherichia/Shigella* have been reported in legume-fed rats or pigs [14,31,33]. Several genera belonging to the *Enterobacteriaceae* Family have been considered fatal pathogens because of their resistance to antibiotics and their implication in a variety of diseases [34]. Sequencing and LEfSe analysis revealed that the genera most likely to explain differences between CPI and LI were *Ruminococcus, Prevotella* and other genera from *Prevotella* for CPI; *Methanobrevibacter* spp. (*Archaea*), and genera from *Desufovibrionaceae* and *Alphaproteobacteria* for LI. Finally, the organisms most likely to explain the differences between animal (milk) and vegetable (legume) proteins were genera from the order *Bacteroidales, Parabacteroides* spp. and *Phascolarctobacterium* spp. for the animal proteins; genera from the family S24_7 and from the order *Clostridiales, Prevotella* spp., genera from *Bacteroidaceae, Clostridiaceae, Cyanobacteria* and *Coriobacteriaceae* for the vegetable proteins. Higher *Prevotella* spp. in the legume PI groups with respect to the milk protein groups (Table 3) is also in line with previous reports with legume-fed rats, where cowpea-based diets modulated the intestinal microbiota to the Prevotella enterotype, which has been linked to lower colon inflammation markers [35,36]. In addition, the effect found on *Methanobrevibacter* spp. (see Table 3 and Figure 3) by the LI diet is remarkable and, to our knowledge, not previously reported. Methanogens have been shown to participate in the reduction of methyl compounds (mono, di and trimethylamine (TMA)) to produce methane. This could be important, considering that TMA, produced through the metabolism of choline and L-carnitine by gut microorganisms, is subsequently oxidized in the liver into the proatherogenic trimethylamine oxide (TMAO). Based on this consideration, it has been hypothesized that dietary supplementation with so-called “Archaebiotics” could prevent cardiovascular diseases in at-risk subjects [37]. Therefore, the mostly beneficial modulation of intestinal microbiota found in legume-fed animals is likely to be due, at least in part, to the protein fraction of the meal. However, it is important to indicate at this point that the current work has the limitation that only semisynthetic diets with highly purified proteins were used here. The putative effects of the different proteins in a complex diet with a variety of ingredients would also be modulated by interactions among dietary chemical fractions, microbial degradation of other dietary fractions, microbial cross feeding phenomena, etc. The present investigation was just a first approach to the issue of understanding, in a design as simple as possible, the behavior of some dietary proteins in the absence of other dietary components.

Finally, the discriminant analysis (Figure 4) of the PICRUSt analysis showed that the functionality of the intestinal microbiome in rats fed the diets differing in the type of protein differed significantly (*p* < 0.05). Also, most functions of the caecal microbiota were differentially enriched in the LA group (Figure S1). The exceptions were Methanogenesis_PWY, which was enriched in the LI group (in line with the increased *Methanobrevibacter* spp. in this group), and PWY_3781 which was enriched in the CAS group. It is noteworthy that this differentially enriched functionality observed in the LA group was accompanied by a lower richness (Table 4) in this group. An enriched functionality is, therefore, not necessarily linked to a higher bacterial richness. The LEfSe analysis (Figure 5) of predicted functions outlined a number of functions discriminating animal (milk) from vegetable (legume PIs) but also CAS from LA.

## 5. Conclusions

Both qPCR and Illumina sequencing analysis revealed significant differences in intestinal microbiota composition and functionality as a consequence of feeding rats with diets that differed only in their constituent proteins. Lower richness values for the LA diet as compared to the other groups including CAS were observed. According to current research, and in the absence of other dietary components, LA appears to induce a generally healthier microbiota composition than CAS and would be then preferable in high-protein diets. Also, Methanogens (*Methanobrevibacter* spp.) and Methanogenic_PWY functionality have been outlined in the LI group, which might be relevant in cardiovascular at-risk subjects. LEfSe analysis of predicted functions (as determined by PICRUSt) discriminated animal (milk) from vegetable (legume PIs) proteins, but also CAS from LA. The mostly beneficial modulation of intestinal microbiota generally found with legume-based diets in vivo is likely to be due, at least in part, to the constituent protein fraction of the meal.

## Figures and Tables

**Table 1:** AAs composition (mg/g) of the dietary proteins used (as in [4]).

	LA ^1^	CAS	CPI	LI
Asp	98.4	58.9	92.7	93.7
Glu	142.9	184.1	123.3	185.3
Ser	39.6	49.1	39.1	43.9
Gly	18.9	16.5	27.9	34.6
His	17.2	27.5	19.1	21.4
Arg	28.5	37.4	58.9	80.4
Thr	45.3	39.6	26.4	30.3
Ala	51.2	32.6	33.5	30.7
Pro	51.7	82.8	38.9	44.5
Tyr	38.1	49.0	24.8	38.7
Val	55.2	53.3	38.5	38.9
Met	15.4	24.5	15.9	6.8
Cys	23.3	3.4	12.3	10.0
Ile	54.0	40.9	40.5	44.7
Leu	118.8	74.9	68.7	70.9
Phe	37.7	46.9	55.8	42.4
Trp ^2^	16.7	10.2	5.5	8.8
Lys	89.3	97.9	50.4	41.6

^1^ LA, lactalbumin; CAS, casein; CPI, chickpea protein isolate; LI, lupin protein isolate; ^2^ Literature values [12,13].

**Table 2:** ANOSIM (distance measure: Bray–Curtis, Bonferroni corrected *p*-values) of Illumina sequencing results at different taxonomic levels of samples from the caecal bacterial community of rats fed diets based on milk (LA, CAS) or legume protein isolates (CPI, LI) as the only protein source.

	Diet ^1^			
	LA	CAS	LI	CPI
**Family**				
LA	0	0.0003	0.0014	0.0002
CAS		0	0.0076	0.0009
LPI			0	0.0438
CPI				0
**Genus**				
LA	0	0.0007	0.0006	0.0004
CAS		0	0.0049	0.0003
LPI			0	0.001
CPI				0

^1^ LA, la ctalbumin; CA S, casein; CPI, chickpea protein isolate; LPI, lupin protein isolate.

**Table 3:** Proportions of Illumina sequencing reads at different taxonomic levels of the caecal bacterial community of rats fed diets based on milk (LA, CAS) or legume protein isolates (CPI, LI) as the only protein source. “f__”, “g__” and “s__” indicate unknown family, genus and species, respectively.

	Diet ^1^				
	LA ^2^	CAS	CPI	LI	*p*-Values ^3^
Phylum					
**Euryarchaeota**	2 ^b^	0 ^b^	4 ^b^	103 ^a^	<0.0001
Actinobacteria	80	216	193	108	0.216
**Bacteroidetes**	2196 ^a^	1208 ^c^	1658 ^b^	1393 ^bc^	<0.0001
Firmicutes	1480 ^b^	1562 ^ab^	1470 ^b^	1717 ^a^	0.058
**Proteobacteria**	177 ^b^	285 ^a^	183 ^b^	236 ^ab^	0.027
**Spirochaetes**	0 ^b^	661 ^a^	619 ^a^	420 ^a^	<0.0001
Family					
**Methanobacteriaceae**	2 ^b^	0 ^b^	5 ^b^	100 ^a^	0.000
Bifidobacteriaceae	52 ^b^	216 ^a^	165 ^ab^	113 ^ab^	0.103
Bacteroidales;f__	93 ^a^	76 ^a^	48 ^a^	39 ^a^	0.144
**Bacteroidaceae**	667 ^a^	179 ^b^	302 ^b^	324 ^b^	0.037
Porphyromonadaceae	45	56	37	31	0.184
**Prevotellaceae**	5 ^c^	0 ^c^	94 ^a^	35 ^b^	0.002
Bacteroidales;f__S24-7	670	660	802	792	0.592
[Paraprevotellaceae]	386 ^a^	177 ^ab^	196 ^ab^	129 ^b^	0.070
Cyanobacteria;f__	0	7	9	21	0.220
Lactobacillaceae	12	41	14	33	0.175
**Clostridiales;f__**	70 ^b^	92 ^ab^	152 ^a^	142 ^a^	0.025
**Clostridiaceae**	98 ^a^	12 ^b^	20 ^b^	25 ^b^	0.015
**Lachnospiraceae**	342 ^ab^	139 ^b^	331 ^ab^	582 ^a^	0.013
**Ruminococcaceae**	773 ^b^	996 ^a^	745 ^bc^	619 ^c^	0.000
Veillonellaceae	90 ^b^	156 ^a^	90 ^b^	84 ^b^	0.007
Erysipelotrichaceae	77	52	102	116	0.204
Alphaproteobacteria;o__RF32;f__	5	33	15	34	0.241
Alcaligenaceae	70	56	45	57	0.595
**Helicobacteraceae**	51 ^c^	173 ^a^	96 ^bc^	115 ^ab^	0.004
Enterobacteriaceae	6 ^a^	12	6	4	0.334
Spirochaetaceae	0 ^b^	6 ^a^	609 ^a^	428 ^a^	<0.0001
Genera					
**Archaea;__Methanobrevibacter**	00 ^b^	0 ^b^	2 ^b^	71 ^a^	<0.0001
Bifidobacterium	53 ^a^	159 ^a^	168 ^a^	97 ^a^	0.103
Bacteroidales;f__;g__	104 ^a^	75 ^ab^	40 ^b^	60 ^ab^	0.134
**Bacteroides**	523 ^a^	193 ^b^	204 ^b^	300 ^b^	0.013
**Parabacteroides**	46 ^a^	55 ^a^	32 ^ab^	18 ^b^	0.028
**Prevotella**	0 ^b^	0 ^b^	115 ^a^	35 ^b^	<0.0001
Bacteroidales;f__S24-7;g__	676	663	803	784	0.642
**[Prevotella]**	1 ^b^	149 ^a^	191 ^a^	25 ^b^	<0.0001
**Chloroflexi;c__S085;o__;f__;g__**	24 ^b^	60 ^a^	3 ^b^	12 ^b^	0.013
Cyanobacteria;c__4C0d;g__	0 ^b^	7 ^ab^	9 ^ab^	22 ^a^	0.143
Lactobacillus	12 ^a^	42 ^a^	14 ^a^	33 ^a^	0.128
**Clostridiales;f__;g__**	63 ^b^	94 ^b^	152 ^a^	143 ^a^	0.002
Lachnospiraceae;g__	75 ^a^	96 ^a^	204 ^a^	259 ^a^	0.256
Blautia	57 ^a^	21 ^b^	32 ^ab^	17 ^b^	0.069
Coprococcus	30 ^ab^	8 ^b^	31 ^ab^	47 ^a^	0.115
**Roseburia**	137 ^a^	10 ^b^	23 ^b^	17 ^b^	<0.0001
**Ruminococcaceae;g__**	306 ^bc^	240 ^c^	331 ^ab^	387 ^a^	0.006
Oscillospira	55	51	51	60	0.910
**Ruminococcus**	452 ^b^	651 ^a^	474 ^b^	226 ^c^	<0.0001
**Phascolarctobacterium**	89 ^b^	147 ^a^	91 ^b^	83 ^b^	0.016
Allobaculum	73	49	97	114	0.197
Alphaproteobacteria;o__RF32;f__;g_	3 ^b^	32 ^ab^	15 ^ab^	38 ^a^	0.105
Sutterella	69	56	45	58	0.626
Helicobacteraceae;g__	22	24	54	51	0.235
**Helicobacter**	29 ^b^	136 ^a^	43 ^b^	30 ^b^	<0.0001
**Treponema**	9 ^c^	644 ^a^	667 ^a^	436 ^b^	<0.0001

^1^ LA, lactalbumin; CA S, casein; CPI, chickpea protei n isolate; LPI, lupin protein isolate. ^2^ Values are means of 6 animals per group. ^3^ Values with different superscript letters differ significantly. Groups with significant differences have been highlighted in bold.

**Table 4:** Diversity indices Simpson, Shannon, Evenness and Chao1 at different taxonomic levels of sequencing analysis on proportions of samples from the caecal bacterial community of rats fed diets based on milk (LA, CAS) or legume protein isolates (CPI, LI) as the only protein source.

	Diet ^1^				
	LA ^2^	CAS	LI	CPI	*p*-Values ^3^
**Family**					
Simpson	0.829 ^c^	0.842 ^bc^	0.865 ^a^	0.862 ^ab^	0.003
Shannon	2.070 ^c^	2.200 ^b^	2.330 ^a^	2.275 ^ab^	0.000
Evenness	0.480	0.482	0.505	0.501	0.743
Chao1	16.571 ^c^	18.929 ^b^	20.375 ^a^	19.750 ^ab^	<0.0001
**Genus**					
Simpson	0.854	0.874	0.877	0.880	0.136
Shannon	2.301 ^b^	2.438 ^a^	2.500 ^a^	2.469 ^a^	0.008
Evenness	0.479	0.498	0.487	0.493	0.933
Chao1	21.143 ^c^	23.286 ^b^	25.125 ^a^	24.167 ^ab^	<0.0001

^1^ LA, lactalbumin; CAS, casein; CPI, chickpea protein isolate; LPI, lupin protein isolate. ^2^ Values are means of 6 animals per group. ^3^ Values with different superscript letters differ significantly.

## Data Availability

Data are contained within the article and supplementary materials.

## Supplementary Materials

The following supporting information can be downloaded at: https://www.mdpi.com/article/10.3390/nu16010149/s1, Figure S1. ANOVA of PICRUSt functional analysis after SEMPER (50% dissimilarity) analysis, using the default parameters (LDA score = 2). Table S1. SIMPER analysis at different taxonomic levels of caecal bacterial community in rats fed diets based in milk (LA, CAS) or legume proteins isolates (CPI, LI) as the only protein source. “f__”, “g__” and “s__” indicate unknown Family, Genus and Species, respectively.

## Abbreviations

AA, amino acids; CAS, casein; CPI, chickpea protein isolate; LA, lactalbumin; LI, lupin protein isolate; PI, protein isolate; SCFA, short chain fatty acids.
