# Supplementary material for: Dietary Milk or Isolated Legume Proteins Modulate Intestinal Microbiota Composition in Rats
Source: Nutrients. 2024 Jan 2;16(1):149. doi: 10.3390/nu16010149 (PMC10781060; doi:10.3390/nu16010149)
Supplement: Supplementary file 1 [file nutrients-16-00149-s001.zip › nutrients-2793496-supplementary.pdf]

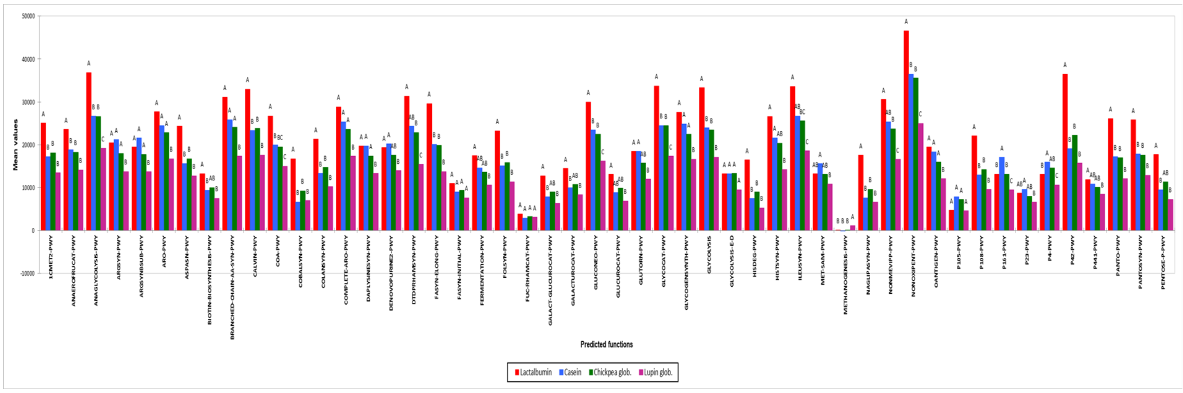

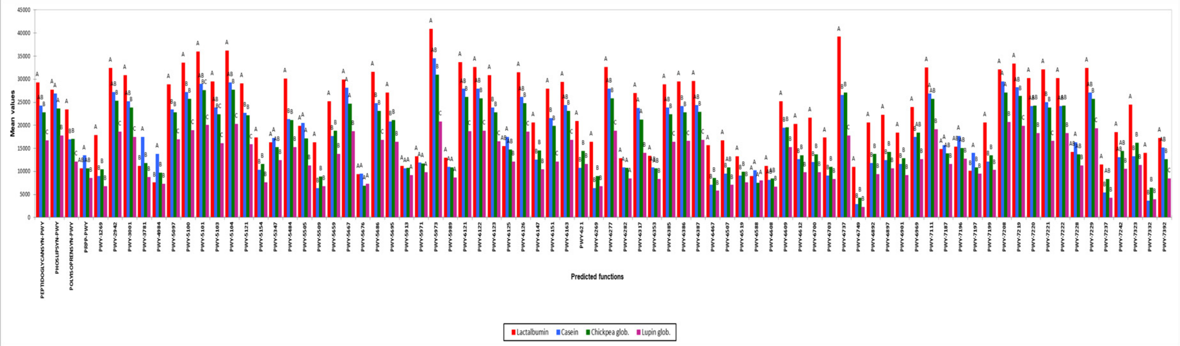

Figure S1. ANOVA of PICRUSt functional analysis after SEMPER (50% dissimilarity) analysis, using the default parameters (LDA score = 2).

Table S1. SIMPER analysis at different taxonomic levels of caecal bacterial community in rats fed diets based in milk (LA, CAS) or legume proteins isolates (CPI, LI) as the only protein source. “f__”, “g__” and “s__” indicate unknown Family, Genus and Species, respectively.

**Family**	Average dissimilarity	Contribution %	Cumulative %
Bacteroidaceae	4.945	12.69	12.69
Spirochaetaceae	4.793	12.3	25
Lachnospiraceae	4.466	11.46	36.46
Bacteroidales;f__S24-7	3.525	9.047	45.51
Ruminococcaceae	3.213	8.247	53.75
[Paraprevotellaceae]	2.518	6.463	60.22
Bifidobacteriaceae	1.673	4.294	64.51
Prevotellaceae	1.57	4.03	68.54
Clostridiales;f__	1.384	3.553	72.09
Helicobacteraceae	1.022	2.623	74.72
Bacteroidales;f__	1.014	2.603	77.32
Erysipelotrichaceae	0.9098	2.335	79.66
Clostridiaceae	0.78	2.002	81.66
Methanobacteriaceae	0.6966	1.788	83.45
Lactobacillaceae	0.6795	1.744	85.19

Veillonellaceae	0.6602	1.695	86.88
Alphaproteobacteria;f__	0.6456	1.657	88.54
Chloroflexi;f__	0.6297	1.616	90.16
Alcaligenaceae	0.5317	1.365	91.52
Cyanobacteria;f__	0.4922	1.263	92.79
Porphyromonadaceae	0.468	1.201	93.99
Enterobacteriaceae	0.3877	0.9953	94.98
**Genus**			
Treponema	4.893	13.05	13.05
Bacteroidales;f__S24-7;g__	3.917	10.45	23.5
Ruminococcus	3.899	10.4	33.91
Lachnospiraceae;g__	3.263	8.706	42.61
Bacteroides	3.012	8.036	50.65
[Prevotella]	2.058	5.492	56.14
Bifidobacterium	1.598	4.264	60.41
Ruminococcaceae;g__	1.391	3.712	64.12
Roseburia	1.007	2.686	66.8
Helicobacter	1.006	2.684	69.49
Allobaculum	0.9261	2.471	71.96
Clostridiales;f__;g__	0.8719	2.326	74.28
Bacteroidales;f__;g__	0.8552	2.282	76.57
Phascolarctobacterium	0.6782	1.81	78.38
Lactobacillus	0.6111	1.631	80.01
Prevotella	0.5774	1.541	81.55
Chloroflexi;g__	0.5226	1.394	82.94
Alphaproteobacteria;g__	0.5188	1.384	84.33
Sutterella	0.495	1.321	85.65
Helicobacteraceae;g__	0.4872	1.3	86.95
Blautia	0.4147	1.106	88.05
Coprococcus	0.3817	1.018	89.07
Oscillospira	0.378	1.009	90.08
Archaea;__Methanobrevibacter	0.3698	0.9865	91.07
Parabacteroides	0.3682	0.9825	92.05
Cyanobacteria;g__	0.3319	0.8856	92.93

[1]LA, lactalbumin. CAS, casein; CPI, chickpea protein isolate; LI, lupin protein isolate. [2] Values are means of 6 animals.
